# The reasons behind medicine shortages from the perspective of pharmaceutical companies and pharmaceutical wholesalers in Finland

**DOI:** 10.1371/journal.pone.0179479

**Published:** 2017-06-28

**Authors:** Kati Heiskanen, Riitta Ahonen, Risto Kanerva, Pekka Karttunen, Johanna Timonen

**Affiliations:** 1 School of Pharmacy/Social Pharmacy, Faculty of Health Sciences, Kuopio Campus, University of Eastern Finland, Kuopio, Finland; 2 Espoo I Tapiola Pharmacy, Espoo, Finland; 3 Siilinjärvi I Pharmacy, Siilinjärvi, Finland; Jagiellonian University, POLAND

## Abstract

The aim of this study was to explore the reasons behind medicine shortages from the perspective of pharmaceutical companies and pharmaceutical wholesalers in Finland. The study took the form of semi-structured interviews. Forty-one pharmaceutical companies and pharmaceutical wholesalers were invited to participate in the study. The pharmaceutical companies were the member organizations of Pharma Industry Finland (PIF) (N = 30) and the Finnish Generic Pharmaceutical Association (FGPA) (N = 7). One company which is a central player in the pharmaceutical market in Finland but does not belong to PIF or FGPA was also invited. The pharmaceutical wholesalers were those with a nationwide distribution network (N = 3). A total of 30 interviews were conducted between March and June 2016. The data were subjected to qualitative thematic analysis. The most common reasons behind medicine shortages in Finland were the small size of the pharmaceutical market (29/30), sudden or fluctuating demand (28/30), small stock sizes (25/30), long delivery time (23/30) and a long or complex production chain (23/30). The reasons for the medicine shortages were supply-related more often than demand-related. However, the reasons were often complex and there was more than one reason behind a shortage. Supply-related reasons behind shortages commonly interfaced with the country-specific characteristics of Finland, whereas demand-related reasons were commonly associated with the predictability and attractiveness of the market. Some reasons, such as raw material shortages, were considered global and thus had similar effects on other countries.

## Introduction

The availability of medicines is regarded as good when patients have access to affordable, good-quality medicinal treatment when and where needed [1–2]. According to WHO, good availability of medicines is a priority health matter [3]. However, in recent years, good availability of medicines has not been achieved and medicine shortages have been experienced globally [4–5]. A medicine shortage can be defined as “a drug supply issue requiring a change that impacts patient care and requires the use of an alternative agent” [5].

Medicine shortages cause several issues related to patient care and patient safety [6–16]. They may result in medication errors, inefficiency in medical care, and side effects [7–12, 14]. Furthermore, patients may suffer from delays or may not receive the recommended treatment at all [9–10]. Shortages may also add to the workload of physicians and pharmacists [6, 9–10, 12–14] and increase the cost of medical care [9–11].

Research data on medicine shortages is still limited [4–11, 15], although some research findings have been published on the frequency of medicine shortages [6, 9, 15]. According to a Finnish study, 80 percent of community pharmacies faced medicine shortages daily or almost daily during the 27-day study period [6]. In another report, 63 percent of hospital pharmacists reported medicine shortages on a weekly, or even daily, basis during the past year [15]. Correspondingly, more than half of all health care practitioners reported shortages frequently or always during the past year [9]. Furthermore, the frequency of shortages impacting patient care has increased during recent years [9, 15]. Information on the frequency of shortages is, however, fragmented and is mainly presented at summit meetings and in reports of federations in the pharmaceutical field [4–5, 8, 11]. In addition, there are some earlier research publications on the impacts of shortages on patient safety, patient care and increased workload of health care professionals [6–8, 10, 13, 15], demonstrating that the negative effects of shortages have been recognized.

Current research shows that the reasons behind medicine shortages are often not determined. In a study on shortages in Finnish community pharmacies, only 11 percent of the pharmacies were informed of the reasons behind medicine shortages [6]. In a longitudinal study of emergency departments in the United States, almost half of all shortages were due to unknown reasons [15]. In a Canadian study, 83 percent of health care practitioners had little or no information about the cause of the drug shortage [9]. Multiple reasons, such as manufacturing issues, regulatory issues or economic factors, have been suggested to underlie medicine shortages [4–5, 17–18]. However, to the best of the authors’ knowledge, only a few research publications have appeared on the reasons behind medicine shortages [9, 19–22]. Earlier research emphasizes the need for a study that quantifies and identifies the reasons behind medicine shortages more thoroughly [21]. The aim of this study was to explore the reasons behind medicine shortages from the perspective of pharmaceutical companies and pharmaceutical wholesalers in Finland.

## Study context

Operational environment in the pharmaceutical market varies between countries. Study context introduces the principles of pharmaceutical market and the medicine distribution network in Finland insofar as necessary to understand the country-specific characteristics of and the reasons behind medicine shortages in Finland that are discussed in this study.

In 2015, total sales of pharmaceuticals at wholesale prices were approximately €2.2 billion in Finland [23]. The Finnish market share is approximately 0.3% of the global, and approximately 1.2% of the European pharmaceutical market [24]. The pharmaceutical market is strictly regulated at national and European levels [25–26].

The medicines sold in Finland come mainly from abroad, most of them from other EU countries [24]. In 2016, a total of 38 pharmaceutical companies had operating licenses and activities in Finland [27]. Of these, three manufactured pharmaceuticals in Finland [28], while there were also a few contract manufacturers.

The wholesale distribution of medicines in Finland is based on a single-channel system [29]. The pharmaceutical manufacturer makes an exclusive distribution contract covering all its products with a wholesaler, and pharmacies or hospitals can acquire a certain pharmaceutical product only through the particular wholesaler. In 2016, there were three pharmaceutical wholesalers with a nationwide distribution network in Finland.

Privately owned community pharmacies have exclusive entitlement in selling prescription and over-the-counter medicines (excluding nicotine replacement therapy) to the public. In 2016, there were 613 community pharmacies, 185 subsidiaries and two university-owned pharmacies in Finland [30]. University-owned pharmacies operate in the same way as privately owned community pharmacies. The pharmaceutical network in Finland has wide coverage, as there is at least one pharmacy in almost every municipality [31]. Obligatory generic substitution (GS) and a reference price system (RPS) are in use in Finland [32–33].

Finland has 24 hospital districts, each of which belongs to one of the five expert responsibility areas (ERAs) responsible for specialized care [34]. Hospital trade, meaning trade and purchasing by the hospital districts and ERAs, is organized via obligatory competitive tender procedures [35]. Each ERA has its own competitive tender procedure. The pharmaceutical companies first list the medicines they offer. The contracts are then awarded according to statutory principles that strongly emphasize the price and overall economic efficiency of the medicines [36]. After the tender, the ERAs reserve the medicines for the use of the whole hospital district from the winning bidder for a specified period. The pharmaceutical company awarded the contract agrees to supply the medicines concerned as and when needed. The hospital districts, however, are not obliged to purchase all the medicines they have reserved, only those they actually need during the period concerned.

## Material and methods

The study was conducted as semi-structured interviews [37] with pharmaceutical companies and pharmaceutical wholesalers in spring 2016. Semi-structured interview was selected as the study method as it translates the perspectives and the experiences of the interviewees well. It is also well suited to topics that are rarely studied. Semi-structured interviews have previously been conducted with representatives of pharmaceutical wholesalers in studying the implementation of generic substitution in Finland [33].

A total of 41 pharmaceutical companies and pharmaceutical wholesalers were invited to participate in the study. The pharmaceutical companies were member organizations of Pharma Industry Finland (PIF) (N = 30) and the Finnish Generic Pharmaceutical Association (FGPA) (N = 7). PIF is an interest group of research-based pharmaceutical companies, while FGPA represents generic pharmaceutical companies operating in the pharmaceutical market in Finland [38–39]. Additionally, one pharmaceutical company which is a central player in the pharmaceutical market in Finland but does not belong to PIF or FGPA was invited. The pharmaceutical wholesalers were those with a nationwide distribution network (N = 3).

The chief executive officers or the country managers of the companies first received information about the study via e-mail. For each company, a person with the greatest familiarity with the availability of medicines and medicine shortages, e.g. the chief executive officer or the responsible pharmacist of the company, was invited to be interviewed. The companies who agreed to participate gave their written consent to participate via e-mail. Once this consent had been given, the date and time for the interview were set.

The interview guide (Table 1) consisted of three main topics. The guide was developed based on the literature [4–5] and the authors' expertise and experience, and was piloted in two pharmaceutical companies. No adjustments were made to the form, and the pilots were included in the study material. The interviewees received an interview guide one week before the interview.

**Table 1:** The interview guide.

Main topic	Sub questions of the main topic
The pharmaceutical market in Finland	Are there some characteristics in the pharmaceutical market in Finland that differ in comparison to other countries? Please describe these characteristics. How do these characteristics affect the availability of medicines in Finland? Are some of these characteristics especially problematic? Why?
The pharmaceutical distribution chain in Finland	Are there some characteristics in the pharmaceutical distribution chain in Finland that differ in comparison to other countries? Please describe these characteristics. How do these characteristics affect the availability of medicines in Finland? Are some of these characteristics especially problematic? Why?
The reasons behind medicine shortages	What are the most crucial reasons behind medicine shortages in Finland? Are some of these reasons especially problematic? Why? What are the most crucial reasons behind medicine shortages in your company? Which of the reasons behind the medicine shortages mentioned are global? Why? What suggestions can you make that would reduce medicine shortages and/or improve the availability of medicines in Finland? How about globally?

The interviews were conducted by telephone (KH) between March and June 2016. Of the 41 invited companies, 30 agreed and four declined to participate. Seven companies did not reply to the invitation at all. The study material consisted in total of 30 interviews, of which 27 were conducted in the pharmaceutical companies and three in pharmaceutical wholesalers. The number of interviews was considered adequate, because the data was saturated, meaning the it began to repeat itself [40]. Each company had one interviewee, the exceptions being two companies who wished to have two interviewees to represent different operations related to the availability of medicines in the company. From a total of 32 interviewees, 20 were women and 12 men. The average length of the interviews was 34 minutes (range 21–58 minutes). All representatives gave their consent for the interview to be recorded.

The recorded interviews were transcribed word for word. Two interviewees wished to read their interview transcripts, but these were returned with no revisions suggested. The data were subjected to qualitative thematic analysis [41–43]. In thematic analysis, the first step is to become familiar with the data and then to mark any similarities and preliminary topics in the data [42–43]. Similar data is then tentatively coded into final topics that are later divided into classifications and further into meaningful categories. In this study, the analysis was started by reading the interviews. The preliminary topics that arose from the interview texts were then marked in the texts. Analysis units were either phrases, sentences or single words describing an idea relating to the study question. The preliminary topics were then divided into final topics. Final topics were formed by combining similar preliminary topics. Each final topic contained one to five preliminary topics. Once the final topics were formed, the analysis was continued based on final topics only. Final topics were then divided into classifications and further into categories. Classifications and categories were formed as reported in the literature [4–5] (Table 2). The analysis was conducted by one researcher (KH), although it was continuously discussed with other research group members. In order to increase the reliability of the analysis, quantifications and tabulations were also used [43]. Quantifications were concluded from the spontaneous mentions of each reason behind medicine shortages in the 30 interviews. Each reason was quantified once per interview, regardless of any multiple mentions in the same interview.

**Table 2:** Suggested reasons behind medicine shortages based on the literature [4–5] and the categories and classifications formed in this study.

	Suggested reasons behind medicine shortages based on the literature	Categories and classifications formed in this study
Supply side	Batch recall and deficient quality assurance Information management Inventory and stock practices Manufacturing issues Raw and bulk material issues Regulatory issues Wholesale and distribution issues	Communication and monitoring Logistics and distribution issues Manufacturing issues Pharmaceutical market structure Regulatory issues
Demand side	Changes in demand Non-traditional demand	Changes in demand Non-traditional demand

The study setting and research process were in accordance with the local and national ethical instructions for research of Finnish Advisory Board on Research Integrity [44]. This study did not contain any features presented by the Finnish Advisory Board on Research Integrity, that required submitting the research to ethical review [45], and therefore we did not seek for ethical statement.

## Results

The data were divided into two categories: “supply-related reasons behind medicine shortages” and “demand-related reasons behind medicine shortages” (Table 3). In the next sections, the most common reasons behind the shortages mentioned in the interviews (≥18/30) are described in detail. Citations of the interviewees on the most common reasons behind the shortages are illustrated in Table 4. The citations were selected to represent the best practical illustrations of each reason behind the shortages.

**Table 3:** Reasons behind medicines shortages from the perspective of pharmaceutical companies and pharmaceutical wholesalers in Finland.

Reason				n^1^
**Supply-related reasons**				
	Pharmaceutical market structure			
		Small market size		29
		Long delivery time^a^		23
		Dependence on foreign manufacturing		22
		Limited number of operating companies		19
		Small language area with two native languages and specific packaging requirements^b^		16
		Mandatory reserve supplies^c^		13
	Logistics and distribution issues			
		Small stock size^d^		25
		Geographical position and long distances		12
		Error in distribution		5
	Manufacturing issues			
		Long or complex production chain		23
		Raw material shortage		21
		Production issues		19
		Capacity issues		18
		Quality and process issues		16
		Changes in marketing authorization holder		13
		Changes at production site		7
		Natural disaster		3
	Regulatory issues			
		Tightened regulation by the authorities		16
	Communication and monitoring			
		Inadequate communication and monitoring and human errors		3
**Demand-related reasons**				
	Changes in demand			
		Sudden or fluctuating demand		28
	Structure of demand			
		Business decisions		
			Low price level or low profitability in Finland	21
			Prioritization of countries or markets	19
			Prioritization of pharmaceutical companies	6
			Spot trade^e^	5
		Hospital trade and tenders		18
		Reimbursement issues		13
		Generic substitution and reference price system		7
	Non-traditional demand			
		Parallel trade		6
	Perceived limited financial purchasing capability			
		Limited willingness and capability to pay		4

^1^ A total of 30 interviews were conducted. “n” refers to the number of spontaneous mentions of each reason behind medicine shortages in the interviews. Each individual reason was quantified once per interview, regardless of any multiple mentions in the same interview.

^a^ The time elapsed from the pharmaceutical companies ordering the medicine from headquarters or from the manufacturing site to receiving it in Finland may vary from a few months up to 24 months

^b^ There are two native languages in Finland: Finnish and Swedish. Due to this, medicine marketed and sold in Finland has specific package requirements that require the use of both native languages in the packaging

^c^ Pharmaceutical companies are obliged to stock certain groups of medicinal products (such as antibiotics, analgesics and cardiovascular medicines) equivalent to 3, 6 or 10 months’ domestic sales depending on the medicine group

^d^ In any or all parts of the distribution network

^e^ A form of trade where a pharmaceutical company launches a small medicine batch that is sold at a low price and then exits the market without taking long-term responsibility for the availability of the medicine

**Table 4:** Citations of the interviewees on the most common^1^ reasons behind medicine shortages in this study.

Reason behind the shortage	Citation
Long delivery time	“the supply chain, which is usually very long, means delivery times from the point you notice you need the goods to producing the raw materials, producing and analyzing medicines and so on, can easily be six to nine months, in fact, six months is usually the very minimum” (interviewee 22)
Limited number of operating companies	“we have more and more situations in which there are only a few suppliers of some crucial active substance or excipient. That makes it (the supply chain) very vulnerable” (interviewee 5)
Small stock size	“companies are not keen to maintain high stock levels, and new batches are not produced until the previous batch is exhausted. The margin is kept quite small, of course, because money is tied up in stocks” (interviewee 14)
Long or complex production chain	“most of the subcontractors producing raw materials are actually outside Europe, --- though even if they were located in Finland, much of their work is outsourced from elsewhere. These issues may sometimes severely compromise the availability of products” (interviewee 27)
Raw material shortage	“raw material issues are the reasons behind shortages at global and production levels. In some cases, medicines have not been imported into Finland quickly enough --- the manufacturer has not received some raw material or other substance that is necessary in the production of the product in question” (interviewee 21)
Capacity issues	“companies are forced to seek profitability from every step of the process --- which also affects availability --- previously, the companies had more capacity --- nowadays, the pharmaceutical industry generally seeks to maximize production capacity so that no extra capacity is available” (interviewee 5)
Sudden or fluctuating demand	“once our competitor has shortages and they have supply issues, our sales explode and exceed all predicted levels --- we can’t respond to this need” (interviewee 1)
Low profitability or low price level in Finland	“once the competitive tender procedures continue long enough and the rules become strict enough, companies have to start thinking about what they can tender safely” (interviewee 7)
Hospital trade	“one particular feature that definitely affects the availability of medicines is the hospital trade and the length of the contract periods. As contract periods last two years, nowadays even three years, once your medicinal product wins the tender, other suppliers are out for two-three years” (interviewee 8)

^1^ Reasons mentioned in at least 18 of the 30 interviews

### Supply-related reasons behind medicine shortages

#### Pharmaceutical market structure

The structure of the pharmaceutical market in Finland was considered a significant factor affecting medicine shortages. In almost all interviews, the size of the pharmaceutical market in Finland was considered small in comparison to other European countries or the global pharmaceutical market, and thus one reason behind medicine shortages (29/30). The volumes of medicines marketed in Finland were also considered small.

Long delivery time, meaning the time elapsed from when the pharmaceutical companies ordered the medicine from headquarters or from the manufacturing site to when they received it in Finland, was commonly brought up in the interviews (23/30). The delivery time varied from a few months up to 24 months (Table 4). Long delivery times meant that the medicines ordered did not always arrive in Finland in time, resulting in shortages. Furthermore, long delivery times oblige pharmaceutical companies and pharmaceutical wholesalers to predict sales well in advance and place orders well in advance of actual sales.

Dependence on foreign manufacturing was also frequently brought up in the interviews (22/30). Greater domestic manufacturing was considered to reduce the risk of medicine shortages in Finland, especially in global crisis situations. Dependence on foreign manufacturing was also considered to result in long or complex production chains and to lengthen the delivery time of medicines to Finland.

In relation to the pharmaceutical market structure in Finland, the limited number of operating companies was also said to cause shortages (19/30) (Table 4). The limited number of operating companies may be the result of the low-profitability, unattractive market, where only companies with big volumes survive. The limited number of operating companies was experienced as difficult and laborious in terms of both finished-product manufacturing and raw material supply.

#### Logistics and distribution issues

Low stocks, in any or all parts of the distribution network, were considered to cause medicine shortages (25/30) (Table 4). Maintaining low stock levels might be a business decision, as larger stocks cause additional warehousing costs and therefore adversely affect the company’s overall profitability. Warehousing costs in Finland were regarded as higher than in some European countries.

#### Manufacturing issues

In terms of manufacturing issues, the most common reason behind medicine shortages was a long or complex production chain (23/30) (Table 4). Production chains with multiple subcontractors caused medicine shortages, whereas shorter, direct production chains reduced them. This is because controlling the reliability of the production chain is more difficult as the production chain becomes longer or more complex. A long or complex production chain and the use of multiple subcontractors was believed not only to compromise the availability of medicines, but also to increase the risk of quality issues and to raise manufacturing costs.

Shortages of raw material for the production of active substances or other ingredients were another common reason behind medicine shortages (21/30) (Table 4). Raw material shortages were frequently considered a global issue, partly because long or complex production chains result in just a limited number of operating companies. The problem occurs when many finished-product manufacturers rely on subcontractors that in turn are dependent on a certain raw material producer. When the raw material producer faces quality issues in his own production, the raw material is not forwarded to the subcontractors and on to the manufacturers.

Production-related issues were frequently mentioned as one reason for medicine shortages (19/30). These relate to the manufacturing process at the production site and may take the form of a malfunction of the production or packaging line. Like raw material shortages, production issues were regarded as a common reason for global shortages. Production issues were considered to have far-reaching effects on the availability of medicines.

As far as manufacturing issues are concerned, capacity issues were also regarded as a reason behind medicine shortages (18/30) (Table 4). Capacity issues relate to economic pressures affecting pharmaceutical manufacturing. Pressure to meet high economic targets means that production is highly optimized. If something unexpected occurs, production capacity is exceeded and capacity issues arise. Capacity issues may also accumulate due to production issues. Consider a situation where production is temporarily interrupted for several weeks due to a malfunction on the production line. After the production issue has been solved, the manufacturer tries to cover both the orders from the past few weeks in addition to current orders. If the production is optimized for a certain number of orders and that number suddenly increases, production capacity can easily be exceeded. This may cause medicine shortages especially in smaller markets, which are not prioritized because of the limited production capacity.

### Demand-related reasons behind medicine shortages

#### Changes in demand

In almost all interviews, sudden or fluctuating demand was considered a reason for medicine shortages (28/30). Changes in demand were most often caused by regular seasonal fluctuations in sales and by shortages experienced by other pharmaceutical companies, of which the latter are almost impossible to predict. If one company has a dominant market share, such as 70 percent, and faces a shortage, other companies with smaller market shares are unable to cover the sudden rise in demand and will also face shortages.

Hospital trade (see Study context) was also considered to cause abrupt changes in demand. As the period between the awarding of contracts and actual sales is only a few months, the predictability of sales by pharmaceutical companies that tender for hospital trade is poor. Changes in demand caused by hospital trade also affect outpatient care by reducing predictability and complicating orders. In a small country that depends on foreign manufacturing, long delivery times combined with poor predictability may pose a serious threat to the availability of medicines.

#### Structure of demand

Finland’s low pharmaceutical prices and poor profitability were also common reasons for medicine shortages (21/30) (Table 4). Prices and profits in Finland were considered too low compared to other pharmaceutical markets. It was felt that, in some cases, it may be more profitable to opt out of the pharmaceutical market in Finland than to try and cope with limited volumes and profits. In the long run, this restricts the number of operating companies in the pharmaceutical market, and thus complicates shortage issues.

Prioritization of countries or markets was also regarded as a reason behind medicine shortages (19/30). Prioritization may be the result of low prices and poor profitability, the structure of the pharmaceutical market in Finland or a combination of both. It was emphasized that, in order to prevent the shortage situation from worsening in the future, this should be taken into account in political and regulatory decision-making in Finland.

Hospital trade, either directly or indirectly, was also considered to lead to medicine shortages (18/30). The hospital trade, with its long tender times and quick transition from tender decisions to actual sales, was considered a direct cause of shortages. Furthermore, the hospital trade was considered challenging due to the mandatory reserve supply system. In Finland, pharmaceutical companies are obliged to stock certain categories of medicinal products such as antibiotics, analgesics and cardiovascular medicines, equivalent to 3, 6 or 10 months’ domestic sales depending on the category [46]. This also applies to medicines for hospital use and thus hospital tenders. Thus, winning the tender means covering the needs of the whole hospital district within just a few months. In addition, the winning company is obliged to maintain the required stock level in the mandatory reserve supply system.

## Discussion

This study explored the reasons behind medicine shortages from the perspective of pharmaceutical companies and pharmaceutical wholesalers in Finland. The most commonly mentioned reasons for shortages were the small size of the country’s pharmaceutical market, sudden or fluctuating demand, small stock sizes, long delivery times and long or complex production chain. On a more general level, the reasons behind medicine shortages were more often supply-related than demand-related. The reasons behind medicine shortages were often complex and many factors simultaneously contributed to shortages. The results are consistent with earlier interview and literature-based studies [19–22] and with the reasons for shortages suggested at summit meetings and in reports [4–5, 17–18].

According to this study, the structure of the pharmaceutical market is a key determinant of medicine shortages. The most common reason cited for shortages was the small size of the pharmaceutical market, while three of the six most common reasons for shortages were related to the structure of the pharmaceutical market. Our results are supported by earlier research showing that small market size often equates with fewer product introductions and more delisted products in the market [18]. Long delivery times and inflexible supply chains further exacerbate the shortage issue [17]. It is notable that supply-related market structure features, such as small market size and long delivery times, commonly interface with features that are characteristic of Finland. Based on the results of our study, these country-specific characteristics exacerbate medicine shortages and complicate the shortage issue. It should be noted that these characteristics may be typical of other small countries and markets too.

Whereas supply-related reasons behind medicine shortages commonly interface with the specific characteristics of Finland, demand-related reasons are linked to the predictability and attractiveness of the market. Based on the results of this study, changes in demand and business decisions are common reasons for medicine shortages in Finland. Similar results have been published in other European countries [18, 21–22]. According to our study, demand-related shortages may be induced by several factors such as the hospital trade. Demand-related factors affecting medicine shortages are not restricted to Finland, however. Hospital trade and competitive bidding tenders are widely used in European countries [47]. Earlier research shows that, once contracts have been awarded, the market becomes less attractive and less profitable for those companies whose bids were unsuccessful [21–22]. Furthermore, shortages are more likely to occur in countries where only one tenderer is awarded a contract [21, 48]. Thus, the results of our study support the earlier hypothesis that the lack of market attractiveness is a major root cause of medicine shortages [4, 17–18, 49]. This may be due to the fact that the pharmaceutical industry’s operating costs have grown significantly in the past ten years and the industry has become more reluctant to market medicines [21]. This has led to medicine shortages in less attractive markets globally.

In addition to the supply-related and demand-related country-specific characteristics, there are also global determinants affecting the availability of medicines in Finland. Even though this study is an example of a single EU member state, some reasons behind medicine shortages, such as raw material shortages and production issues, were considered to be global and to have similar impacts in other countries. Our results are supported by the report of the European Healthcare Distribution Association, which states that European countries share common underlying causes of medicine shortages [17]. It should be pointed out that most of the companies that participated in this study operate not just in Finland but globally. Therefore, the reasons behind the medicine shortages described in this study are not restricted to Finland and may affect other countries too.

The results of this study are based on the opinions and answers of the representatives of the pharmaceutical companies and pharmaceutical wholesalers in Finland. This is the only way to study the reasons behind medicine shortages more thoroughly, because only pharmaceutical companies and pharmaceutical wholesalers have knowledge of the reasons behind shortages. In Finland, pharmaceutical companies and pharmaceutical wholesalers are obliged to report medicine shortages to Fimea, although the reasons behind shortages are not made public [50]. In some countries, the root causes of shortages may not be announced publicly because of their adverse effect on marketing authorization holders [22]. At European level, shortages that affect or are likely to affect more than one country are reported to EMA and listed in the shortage catalogue [51]. The shortage catalogue lists the affected countries and the reason behind the shortage. However, the catalogue does not provide a comprehensive listing of the shortages [19].

The participation rate of this study was high, as 30 of the invited 41 companies participated. This is comparable with a Belgian interview study on the reasons behind medicine shortages [19]. In addition, the data of 30 companies included most pharmaceutical companies and all pharmaceutical wholesalers with a nationwide distribution network operating in the pharmaceutical market in Finland, reducing the possibility of response rate bias. Furthermore, the data was saturated with 30 interviews. Therefore, further interviews were unlikely to add new information to the data. In the present study, the semi-structured interviews provide a broad appraisal of the reasons behind medicine shortages and the complexity of the shortage issue in Finland.

This study provides new information on medicine shortages from the perspective of pharmaceutical companies and pharmaceutical wholesalers in Finland. According to the report of FIP, country-specific research is needed to provide a more general view of the shortage issue [5]. In the future, more country-specific research from the perspective of pharmaceutical companies and pharmaceutical wholesalers is needed to explore the reasons behind medicine shortages and how to tackle medicine shortages globally.

## Conclusions

The most common reasons behind medicine shortages from the perspective of pharmaceutical companies and pharmaceutical wholesalers in Finland were the small size of the pharmaceutical market, sudden or fluctuating demand, small stock sizes, long delivery time and a long or complex production chain. The reasons behind medicine shortages were more commonly supply-related than demand-related. However, the reasons behind medicine shortages were often complex and there was more than one reason behind a shortage. Supply-related reasons behind shortages commonly interfaced with the specific characteristics of Finland, whereas demand-related reasons were commonly associated with the predictability and attractiveness of the market. Some reasons behind medicine shortages, such as raw material shortages, were considered global and thus have similar effects on other countries.
